# Rapid Turbidimetric Assay to Determine the Potency of Daptomycin in Lyophilized Powder

**DOI:** 10.3390/pharmaceutics7030106

**Published:** 2015-07-09

**Authors:** Eliane Gandolpho Tótoli, Hérida Regina Nunes Salgado

**Affiliations:** School of Pharmaceutical Sciences, Universidade Estadual Paulista, Rod. Araraquara-Jaú, km 1, CEP 14801-902 Araraquara, SP, Brazil; E-Mail: salgadoh@fcfar.unesp.br

**Keywords:** analytical method, daptomycin, microbiological method, new antimicrobial agent, quality control, turbidimetric assay

## Abstract

Daptomycin is an important antimicrobial for clinical practice, mainly because it remains very active against Gram-positive resistant strains, such as methicillin-resistant *Staphylococcus aureus* and vancomycin-resistant enterococci. Development of microbiological methods for the analysis of antimicrobials is highly recommended, since they can provide important information about their biological activities, which physicochemical methods are not able to provide. Considering that there are no studies in the literature describing microbiological methods for the analysis of daptomycin, the aim of this work was to validate a microbiological method for the quantitation of daptomycin by the turbidimetric assay. *Staphylococcus aureus* was used as the test microorganism, and the brain heart infusion broth was used as the culture medium. The validation of the method was performed according to the ICH guidelines, and it was shown to be linear, precise, robust, accurate and selective, over a concentration range of 8.0 to 18.0 µg mL^−1^. Student’s *t*-test showed the interchangeability of the proposed method with a previously-validated HPLC method. The developed turbidimetric method described in this paper is a convenient alternative for the routine quality control of daptomycin in its pharmaceutical dosage form.

## 1. Introduction

The continued emergence and global spread of multi-drug-resistant pathogenic bacteria, especially in hospital settings, require increasingly efficient treatment regimens and the development of new antimicrobial compounds able to overcome these mechanisms of resistance [1]. There are few new antimicrobial agents emerging in the market, and this fact can be due to numerous reasons, such as the high cost to introduce a new molecule in a highly competitive market and the inherent difficulty of identifying new targets for antibiotics. Thus, the emergence of microorganisms that no longer respond to the first-line antimicrobials is increasingly common [2,3].

As a strategy for combating resistant microorganisms, the development of drugs with novel mechanisms of action is required. In this context, a new class of antimicrobials stands out, the cyclic lipopeptides. Daptomycin was the first approved member of this class. This cyclic lipopeptide has a mechanism of action distinct from all other available antimicrobials in clinical practice [4]. The mechanism of action is completely dependent on the physiological levels of calcium in the body. For this reason, it is necessary to add calcium in the culture medium for performing *in vitro* microbiological tests, in order to obtain the satisfactory antimicrobial activity of this drug [5,6].

**Figure 1:**
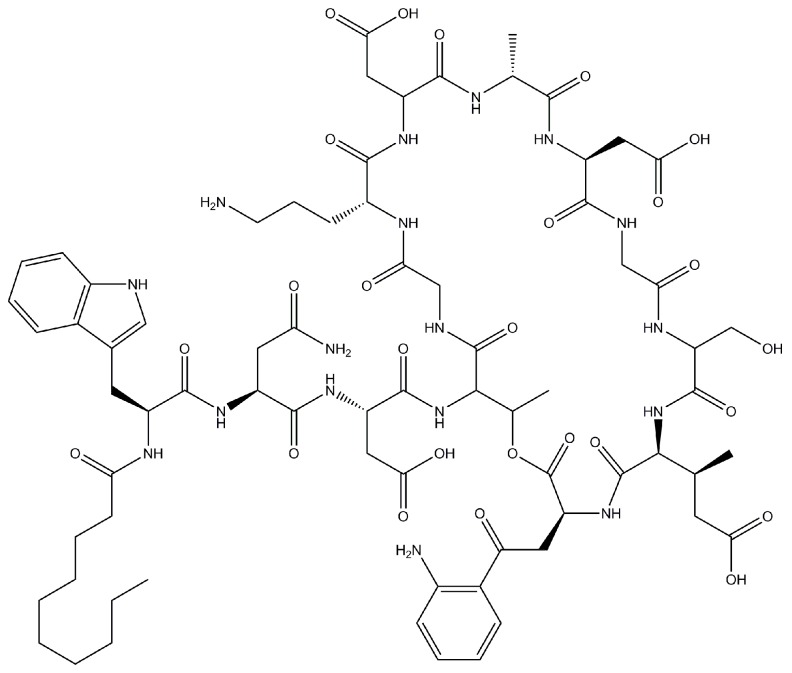
Chemical structure of daptomycin.

Daptomycin (Figure 1) is globally polar and constitutes 13 amino acid residues and an *n*-decanoyl fatty acid chain at the N-terminus [7]. Calcium ions act in two different steps of the mechanism of action of this antimicrobial. In the first step, they gather together the charged amino acids of the molecule on one side and expose its lipophilic tail on the other side, which increases its amphiphilicity. In the second step, the calcium ions favor the oligomerization of daptomycin in micellar structures. These structures, in the presence of negatively-charged membranes, undergo a structural transition that enables the interaction of lipophilic tails of the molecule with the bacterial cell membrane, resulting in its insertion [8]. After that, the molecule of daptomycin undergoes another conformational change that causes a membrane curvature, which allows the leakage of intracellular ions (mainly potassium ions), resulting in depolarization, loss of membrane potential and the consequent inhibition of the synthesis of protein, RNA and DNA [7,8,9].

Daptomycin is an antimicrobial selectively active against aerobic, anaerobic and facultative Gram-positive bacteria [10]. It has activity against β-hemolytic streptococci, methicillin-resistant *Staphylococcus aureus* (MRSA) and vancomycin-resistant enterococci (VRE) [11]. This antimicrobial also shows activity against vancomycin-resistant *Staphylococcus aureus* (VRSA) [12]. It is recommended for the treatment of complicated skin and soft tissues infections, as well as bloodstream infections caused by *Staphylococcus aureus*, including those associated with right-sided infective endocarditis [13]. Considering that the treatment of MRSA and VRE is a challenge for medicine, daptomycin is promising in combating these microorganisms, since resistance to this antibiotic is not frequently reported in the literature. The successful use of daptomycin for the treatment of resistant Gram-positive bacteria still predominates [14,15,16]. For this reason, daptomycin is a very important antimicrobial agent for clinical practice nowadays.

The minimum inhibitory concentration (MIC) of daptomycin against susceptible strains of *Staphylococcus* sp. and *Streptococcus* sp. (A, B, C and G groups) is ≤1 µg mL^−1^ [17] and for *Enterococcus* sp. is ≤4 µg mL^−1^ [18].

The clinical use of daptomycin was approved by the Food and Drug Administration (FDA) in September 2003, in the United States [2,19]. In 2005, daptomycin was also approved by the European Medicines Agency (EMA) in Europe [17]. In 2008, it was introduced into the Brazilian market [19]. Nowadays, it is sold in more than 40 countries worldwide by the pharmaceutical companies Cubist (patent holder), Novartis, Oryx, Sepracor, AstraZeneca, Cardinal Health, TTY Biopharm, Chiron and Hospira [20,21]. It is estimated that more than 2.7 million people have been treated with this antimicrobial agent (between 2003 and 2014) [13]. This fact also highlights the importance of this antimicrobial agent for clinical practice.

In the literature, there are few published studies that show the development of analytical methods for the analysis of daptomycin, and most of them aim at quantifying the drug in biological fluids by high-performance liquid chromatography (HPLC) [22,23,24,25,26,27,28,29] and ultra-performance liquid chromatography (UHPLC) [30,31,32,33,34]. In the same way, there are no monographs for this drug in any official compendium. However, only one study was found in the literature in which an analytical method for the analysis of daptomycin in the pharmaceutical dosage form using HPLC was described [35].

On the other hand, it is known that physical-chemical methods lack the ability to indicate the true biological activity of antimicrobial agents. This fact shows the importance of microbiological methods in the quality control of this type of drug [36,37]. A search through the literature for microbiological methods for the analysis of the potency of daptomycin gave no results.

Considering the importance of daptomycin for the global scene and the lack of literature concerning microbiological methods for the analysis of this antimicrobial, the aim of this work was to propose a rapid turbidimetric method for the analysis of the potency of daptomycin in the dosage form of powder for injectable solution. A high-performance liquid chromatography (HPLC) method, previously developed and validated by us, was chosen as a comparative method.

## 2. Experimental Section

### 2.1. Chemicals

Daptomycin reference standard (DPT RS) (purity of 98.00%) was purchased from Sequoia Research Products Company (Pangbourne, UK). The samples of daptomycin in lyophilized powder for the injectable solution (Cubicin™, Novartis, McPherson, KS, USA) containing 500 mg of the active component were also purchased. The pharmaceutical form contains sodium hydroxide as the excipient. Calcium chloride dihydrate (Synth, Diadema, Brazil) and sodium hydroxide (Vetec, Duque de caxias, Brazil) werealso used.

The culture media used for the tests were a brain heart infusion (BHI) broth (Merck, Germany) and tryptic soy agar (Difco™, Hunt Valley, MD, USA). In order to interrupt the microorganism’s growth, analytical grade formaldehyde (Qhemis™, São Paulo, Brazil) was used. The test microorganism was the *Staphylococcus aureus* ATCC 6538 IAL 2082.

All solutions and mobile phases used for carrying out both microbiological and HPLC methods were prepared using water obtained by a Milli-Q™ Plus apparatus (Millipore™, Darmstadt, Germany). The HPLC mobile phase was prepared using ethanol HPLC grade (JT Baker™, Ecatepec de Morelos, Mexico).

### 2.2. Apparatus

For the microbiological method, the microorganisms were incubated in an ECB Digital 1.2 (Odontobrás, Brodowski, Brazil) oven and in a Shaker incubator, Model MA420 (Marconi, Piracicaba, Brazil). Culture media were sterilized in a vertical autoclave, Model AV (Phoenix Luferco, Araraquara, Brazil), before use. The absorbance values were determined by a spectrophotometer, DU 530 (Beckman Coulter, Pasadena, CA, USA). The analytical curves were constructed using Microsoft Excel (2007) software.

The comparative chromatographic method was carried out using a chromatograph, Model 1525 Waters (Waters Chromatography systems, Milford, MA, USA), which was connected to a Waters 2487 UV/Visible detector and a manual injector Rheodyne Breeze 7725i with a 20-µL loop (Rheodyne Breeze, Cotati, CA, USA).

The following items were also used: H10 analytical scale (Mettler Toledo™, Greifensee, Switzerland); B160 semi-analytical scale (Micronal™, São Paulo, Brazil) and USC2800A ultrasound bath (Unique™, Indaiatuba, Brazil).

### 2.3. Solutions

#### 2.3.1. Preparation of DPT RS Solutions

DPT RS stock solution was prepared by transferring 5.0 mg equivalent of DPT RS to a 50-mL volumetric flask, which was filled with ultrapure water to obtain a concentration of 100 μg mL^−1^. Aliquots of 0.4, 0.6 and 0.9 mL of the DPT RS stock solution were transferred to 5-mL volumetric flasks, the volumes of which were completed with ultrapure water, for obtaining working solutions with concentrations of 8.0, 12.0 and 18.0 µg mL^−1^, respectively named S1, S2 and S3.

#### 2.3.2. Preparation of DPT Sample Solution

The DPT sample stock solution at a theoretical concentration of 100 µg mL^−1^ was prepared. Three vials of DPT powder (commercial available for the preparation of solution for injections) were weighed, and the average weight was calculated. The contents of these vials were mixed. DPT stock solution was prepared by transferring 5.0 mg equivalent of DPT of this mixture to a 50-mL volumetric flask, which was filled with ultrapure water to obtain a concentration of 100 μg mL^−1^ DPT. Aliquots of 0.4, 0.6 and 0.9 mL of this solution were transferred to 5-mL volumetric flasks, the volumes of which were completed with ultrapure water, for obtaining working solutions with concentrations of 8.0, 12.0 and 18.0 µg mL^−1^, respectively named T1, T2 and T3.

#### 2.3.3. Preparation of Calcium Solution

A solution containing 10.0 mg mL^−1^ of calcium was prepared from calcium chloride dihydrate (CaCl_2_·2H_2_O). For this, an amount of 3.66 g of CaCl_2_·2H_2_O was weighed (the equivalent of 1.0 g of calcium), and then, it was transferred to a 100-mL volumetric flask. The volume was completed with ultrapure water.

#### 2.3.4. Preparation of Sodium Hydroxide Solution

A solution containing 1.0 mg mL^−1^ of sodium hydroxide was prepared. For this, NaOH (10 mg) was transferred to a 10-mL volumetric flask. The volume was completed with ultrapure water.

### 2.4. Turbidimetric Assay

#### 2.4.1. Preparation and Standardization of Inoculum

The strain *Staphylococcus aureus* ATCC 6538 IAL 2082 was inoculated in 30 mL of BHI broth and maintained for growth in a microbiological incubator at a temperature of 35 °C ± 2 °C for 24 h before performing the experiment. Thereafter, the inoculum was standardized at 580 nm in a spectrophotometer, in order to obtain a transmittance of 25% ± 2% [38,39]. It is worth remembering that before the whole procedure, the *Staphylococcus aureus* strain was cultivated and kept in tryptic soy agar medium in the freezer.

#### 2.4.2. The Bioassay

First of all, the *Staphylococcus aureus* inoculum was prepared and standardized as described before. Thereafter, in three identical test tubes containing 10 mL of sterile BHI broth, 200 µL of the DPT RS working solutions (S1, S2, S3) and 75 µL of the calcium solution were added. Subsequently, 0.5 mL of the standardized inoculum were also added. The same procedure was carried out for the DPT sample working solutions (T1, T2 and T3). The procedure was performed in triplicate for each DPT concentration. Therefore, twenty test tubes were used, with nine tubes for DPT RS, nine for the DPT samples, one for the positive control (containing BHI broth and inoculum without the addition of DPT) and one for the negative control (containing only the BHI broth).

After the test tubes’ preparation, they were incubated in a shaker, in a water bath, at a temperature of 35.0 °C ± 2.0 °C for 4 h. At the end of the incubation period, the microorganisms’ growth was interrupted by the addition of 0.5 mL of 12% formaldehyde solution in each test tube. The same volume of formaldehyde solution was also added to the negative control tube. Thereafter, the spectrophotometer was reset by the test tube containing the negative control, and the absorbance readings were taken at a wavelength of 530 nm. In each test, the results were statistically analyzed, and the DPT potency was calculated.

#### 2.4.3. Obtaining the Analytical Curve

The analytical curve was constructed using the 3 × 3 parallel line assay design, as recommended by the Brazilian Pharmacopoeia (2010) [38]. For this purpose, the logarithm of the DPT RS working concentrations (8.0, 12.0 and 18.0 µg mL^−1^) *versus* their corresponding average absorbance values were plotted on a graph, with the aid of the Microsoft Excel (2007) software. Three analytical curves were plotted on three different days, and a final analytical curve was obtained with the average of them.

#### 2.4.4. Potency Calculation

The Hewitt equation was used to calculate the DPT potency [40].

### 2.5. Method Validation

The validation of the microbiological method was carried out according to the literature recommendation [38,39,41]. For this purpose, some parameters were determined, such as linearity, precision, accuracy, selectivity and robustness. The limits of detection and quantification are not required for this category of assay.

#### 2.5.1. Linearity

In order to assess the linearity of the method, three analytical curves performed on three different days were analyzed. Each curve was constructed as described before in the Section 2.4.3. The results were analyzed to obtain the equation of the line by the least squares method, and the linearity and parallelism were assessed by analysis of variance (ANOVA).

#### 2.5.2. Precision

Repeatability and intermediate precisions were assessed. For the repeatability, six test tubes containing DPT RS in a concentration of 12.0 μg mL^−1^ were prepared as described in Section 2.4.2. They were analyzed on the same day and at identical working conditions. The relative standard deviation (RSD) between the absorbance values obtained from these six test tubes was calculated and analyzed [41].

Regarding the intermediate precision, it was assessed based on two criteria: inter-assay and between analysts. For the intra-assay precision evaluation, three bioassays were performed on three different days, and the DPT potency was calculated for each day. After that, the relative standard deviation (RSD) between the obtained DPT potency values was calculated and analyzed. In order to assess the between analysts’ precision, two bioassays were performed by two different analysts, and the DPT potency was calculated for each one. Similarly, the relative standard deviation (RSD) between the DPT potency values was calculated and analyzed [41].

#### 2.5.3. Accuracy

The recovery assay was carried out in order to evaluate the accuracy of the microbiological method. For this, known quantities of DPT RS have been added to a synthetic mixture of the drug product excipient [41]. Three different concentrations were assessed, R1, R2 and R3. For this purpose, a stock solution of DPR RS with a concentration of 200 µg mL^−1^ was prepared. An aliquot of 5 mL from this solution was transferred to a 10-mL volumetric flask, and the volume was made up with ultrapure water, to obtain a 100-µg mL^−1^ solution. From this solution, aliquots of 0.8, 1.2 and 1.8 mL were transferred to 10-mL volumetric flasks, and the volumes were completed with ultrapure water, in order to obtain solutions with concentrations of 8.0, 12.0 and 18.0 µg mL^−1^, representing P1, P2 and P3, respectively.

For the preparation of the recovery solutions, an aliquot of 5 mL from the DPT RS stock solution of 200 µg mL^−1^ was transferred to a 10-mL volumetric flask. After that, to the same volumetric flask, 86 µL of the NaOH solution (1.0 mg mL^−1^) were added, in order to simulate the concentration of NaOH that would be in a solution of 100 µg mL^−1^ of daptomycin in lyophilized powder for injectable solution. The volume of the volumetric flask was completed with ultrapure water, resulting in a solution with 100 µg mL^−1^ of DPT RS and 8.6 µg mL^−1^ of excipient (NaOH) (simulated DPT sample solution). From this simulated DPT sample solution, aliquots were transferred to 10-mL volumetric flasks, in order to obtain the recovery solutions, as is described below.

Recovery Test 1: Aliquots of 0.64, 0.96 and 1.44 mL from the simulated DPT sample solution were transferred to 10-mL volumetric flasks, and the volumes were completed with ultrapure water, in order to obtain solutions with the concentrations of 6.4 (R1), 9.6 (R2) and 14.4 (R3) µg mL^−1^, representing a sample of 80% potency.

Recovery Test 2: Aliquots of 0.8, 1.2 and 1.8 mL from the simulated DPT sample solution were transferred to 10-mL volumetric flasks, and the volumes were completed with ultrapure water, in order to obtain solutions with the concentrations of 8.0 (R1), 12.0 (R2) and 18.0 (R3) µg mL^−1^, representing a sample of 100% potency.

Recovery Test 3: Aliquots of 0.96, 1.44 and 2.16 mL from the simulated DPT sample solution were transferred to 10-mL volumetric flasks, and the volumes were completed with ultrapure water, in order to obtain solutions with concentrations of 9.6 (R1), 14.4 (R2) and 21.6 (R3) µg mL^−1^, representing a sample of 120% potency.

Each simulated sample (R1, R2 and R3) was assayed in an independent trial. The percentage of recovery (R%) was calculated by Equation 1:

(1) $$\text{R\%}=\left(\frac{\text{PF}}{\text{TP}}\right)\times 100$$

where PF = the potency found in the recovery samples; TP = theoretical potency.

#### 2.5.4. Selectivity

The selectivity of the method was performed with the aim of showing that the placebo has no antibacterial activity against the test microorganism *S. aureus* under the working conditions. In this way, a placebo solution was prepared consisting of NaOH in the same concentration that it would be in a solution of 18.0 µg mL^−1^ of DPT in lyophilized powder (the higher working concentration). In this case, the concentration is 1.56 µg/mL of NaOH.

The placebo solution (200 μL), standardized inoculum (0.5 mL) and the calcium solution (75 μL) were all added to a test tube containing sterile BHI broth (10 mL). To the same test tube, 0.5 mL of the standardized inoculum and 75 µL of the calcium solution were also added. Then, the turbidimetric assay was carried out as defined under Section 2.4. At the end of the test, the absorbance values provided by the test tube containing the placebo solution were compared to the absorbance values provided by test tubes containing just the culture medium with the inoculum in order to check if the sodium hydroxide caused some inhibition of the bacterial growth. This assay was performed in triplicate.

#### 2.5.5. Robustness

The robustness of the method was evaluated by small modifications, individually, in the following method parameters: concentration of the inoculum, calcium concentration in the culture medium, the wavelength used to determine the results in the spectrophotometer and the period of incubation in the shaker. In this way, bioassays were performed for each modified condition, and the DPT potency was calculated. After that, RSD values were calculated between the responses obtained from the modified and normal conditions.

### 2.6. HPLC Method

An HPLC method previously developed and validated by our research group was used as the comparative method for DPT determination. The chromatographic conditions were: mobile phase consisting of ethanol and water (55:45, *v*/*v*) with pH adjusted to 4.5 with glacial acetic acid; Agilent Zorbax™ C_18_ analytical column (150 × 4.6 mm, 5 µm) (Agilent™, Santa Clara, CA, USA); isocratic elution mode; volume of injection of 20 µL; flow rate of 0.6 mL min^−1^; and UV detection at 221 nm.

### 2.7. Comparison between the Microbiological and Chromatographic Methods

In order to statistically compare the proposed microbiological method with the previously validated HPLC method, six determinations of daptomycin in the pharmaceutical dosage form were performed using both methods. After that, the percentage contents of daptomycin were compared by Student’s *t*-test, at a significance level of 5%, in order to verify whether these results were statistically equivalent.

## 3. Results and Discussion

### 3.1. General Aspects and Method Development

For the development of this work, the method chosen was the turbidimetric assay, mainly due to the advantages that it offers compared to agar diffusion, which is another common microbiological method used for the analysis of antimicrobial agents. The turbidimetric assay is faster than agar diffusion, requiring 4 h to provide the results, while the other demands 24 h of assay [37]. This advantage makes the turbidimetric assay more convenient for routine quality control analysis. Furthermore, the method by agar diffusion presents another limitation, which is the fact that some drugs exhibit difficulty in diffusing through a solid medium. This does not occur in the turbidimetric method, which employs a liquid culture medium.

During the preliminary tests, some microorganisms considered less pathogenic than *S. aureus* were tested before choosing it. The microorganisms tested were: *Kocuria rhizophila* ATCC 9341 IAL 636, *Bacillus atrophaeus* ATCC 9372 IAL 1027 and *Staphylococcus epidermidis* ATCC 12228 IAL 2150. However, it was possible to observe that *K. rhizophila* showed a very slow growth in the tested culture medium (BHI broth) after 24 h of incubation. Regarding the strains of *S. epidermidis* and *B. atrophaeus*, the first one demanded higher concentrations of daptomycin for its growth inhibition (> 60 μg mL^−1^) when compared to *S. aureus*, and the second was not susceptible to daptomycin, even in high concentrations (64 μg mL^−1^). The tested *S. aureus* strain showed satisfactory growth in the culture medium, adequate linearity and reproducible results. For these reasons, this microorganism was chosen for the development of the turbidimetric assay.

Water and phosphate buffer pH 8.0 were tested as diluents. Considering that both showed similar results, water was chosen as the solvent due to economic reasons.

Among the various inoculum concentrations tested (from 4% to 7%), the concentration of 5% showed the best results, due to the appropriate amount of growth of *S. aureus* in relation to the selected concentrations of the antimicrobial. In the same way, among the daptomycin concentrations tested (from 1.0 to 64.0 µg mL^−1^), the best concentrations were 8.0, 12.0 and 18.0 µg mL^−1^, which presented better linearity and accuracy of the results.

Different concentrations of calcium in the culture medium were tested (from 0 to 100 µg mL^−1^), and it was noted that the concentration of 75 mg mL^−1^ resulted in the better activity of daptomycin against *S. aureus*. The assay performed without the addition of calcium in the culture medium showed no inhibition of the microorganism by daptomycin, highlighting the importance of the addition of this substance to the culture medium.

### 3.2. Validation of the Analytical Method

#### 3.2.1. Linearity

By analyzing the DPT RS analytical curve, the method was shown to be linear in the range between 8.0 and 18.0 μg mL^−1^, with a correlation coefficient (*r*) of 0.9995. The obtained equation of the line was *y* = −0.679ln (*x*) + 2.1485.

The linearity of the method was also demonstrated by analysis of variance (ANOVA), which showed that the analytical curve presented no deviation. This conclusion is based on the fact that the *F*_calculated_ for the “quadratic” parameter (0.96) was lower than *F*_critical_ (4.96). The same occurred with the parameter “squared difference” in which *F*_calculated_ (0.26) was also lower than the *F*_critical_ (4.96) value.

Usually, for microbiological methods, it is also necessary to perform an analytical curve for the sample in order to compare it with the analytical curve obtained with the reference standard (parallel-line model). These two curves should be linear and parallel, within the selected working range. These parameters must to be verified by validity tests within a given significance level, which is usually 5% [38,42,43]. This procedure was carried out, and analysis of variance ANOVA showed that the obtained DPT analytical curves (for sample and reference standard) met these requirements.

#### 3.2.2. Precision

Repeatability (intra-assay) and intermediate (inter-assay and between analysts) precisions were evaluated. The RSD value calculated for the repeatability was 4.32%. Inter-assay and between-analyst precisions provided values of 1.48% and 4.08%, respectively. Considering that the RSD values are lower than 5%, the precision of the method was proven, according to the Brazilian legislation for bioanalytical methods [44].

#### 3.2.3. Accuracy

The accuracy was confirmed by the recovery test. In this test, a known amount of DPT RS was added to a solution containing the excipient (spiked placebo). The average recovery was 101.41%, and this fact demonstrates the ability of the method to determine pre-defined contents of DPT with accuracy.

#### 3.2.4. Selectivity

Table 1 shows the results of the selectivity analysis of the method. Comparing the response of the micro-organism with (positive control) and without NaOH, it is possible to observe that the excipient did not interfere in the analysis, since this substance did not present antimicrobial activity (the absorbance values were similar).

**Table 1:** Obtained results for the selectivity analysis of the developed turbidimetric assay.

Analysis	Obtained absorbances for positive control	Obtained absorbances in the test with NaOH
1	0.862	0.830
2	0.913	0.897
3	0.852	0.932
Average	0.876	0.886

#### 3.2.5. Robustness

Table 2 presents the results for the robustness of the method. Considering that all of the varied parameters presented RDS values lower than 5%, the method was shown to be robust.

**Table 2:** Obtained results for the robustness analysis of the developed turbidimetric assay.

Parameter	Investigated range	Daptomycin (g/vial)	Daptomycin (%)	RSD ^1^ (%)
Inoculum concentration (%)	4.8	0.459	91.70	4.22
	5.0 *	0.495	98.94	
	5.2	0.462	92.45	
Calcium concentration (µg mL^−1^)	70	0.519	103.87	2.39
	75 *	0.506	101.17	
	80	0.496	99.11	
Wavelength (nm)	525	0.494	98.79	1.57
	530 *	0.506	101.17	
	535	0.491	98.22	
Shaker incubation time (h)	3 h 50 min	0.484	96.87	3.53
	4 h *	0.506	101.17	
	4 h 10 min	0.520	103.92	

^1^ RDS: relative standard deviation; * standard working conditions.

### 3.3. Comparison between the Microbiological and Chromatographic Methods

A comparison between the developed turbidimetric method with a HPLC method previously developed and validated by our group was performed in order to verify whether these two methods are interchangeable for the analysis of daptomycin in the pharmaceutical dosage form.

Table 3 shows the percentage contents of daptomycin obtained by the microbiological and HPLC methods. Student’s *t*-test performed to compare these data showed that both methods are interchangeable, since the contents of daptomycin obtained by the two methods were statistically equivalent, at a significance level of 5% (*t*_calculated_ = 0.82 < *t*_critical_ = 2.23).

**Table 3:** Values obtained in the determination of daptomycin (DPT) in powder for injectable solution by HPLC and turbidimetric assay.

Parameters	Method	
	HPLC ^a^	TURB ^b^
DPT content (%)	99.88	101.78
	101.90	101.17
	99.10	98.94
	102.90	103.92
	104.37	98.22
	102.85	101.17
Average content (%)	101.83	100.87

^a^ HPLC: high-performance liquid chromatography; ^b^ TURB: turbidimetric assay.

The most interesting aspect of this comparison is that a microbiological method is being compared with a physicochemical method, and both have different characteristics. Microbiological methods, in general, present disadvantages regarding the execution time and the amount of work and materials required. For these reasons, often, these methods are being replaced by physicochemical methods in routine analysis in the quality control of antimicrobials. However, this is not a recommended practice, since microbiological methods provide important information about the biological activity of antimicrobial agents, which physicochemical methods are not able to provide. Frequently, the part of the molecule essential for antimicrobial activity cannot be detected by physical-chemical methods, generating false conclusions about the quality of the product [36,45]. Thus, microbiological assays used to determine the potency of antimicrobial agents still play an essential role in manufacturing processes and quality control of these drugs.

HPLC also presents some advantages and disadvantages when compared to the turbidimetric method. Considering the disadvantages, this technique requires the use of a chromatograph, chromatographic columns and high purity solvents, all costly. In addition, because it is a technique that requires the use of organic solvents, it can harm the environment, in addition to generating costs for the industry to treat this waste. Among the advantages, it is worth highlighting that this technique is rapid, highly selective and ideal for the detection of degradation products and impurities of the analyzed drug [37].

Although the turbidimetric assay is not suitable to determine this kind of impurity and the degradation products, it is an environmentally-friendly method, which does not require the use of organic solvents for its analysis, which is in line with the global trend. Furthermore, it is a method increasingly used and recognized for the analysis of antimicrobial agents [37,46,47,48,49,50].

## 4. Conclusions

A microbiological method has been properly developed and validated and shown to be effective for determining the potency of daptomycin in the pharmaceutical dosage form of lyophilized powder for injectable solution, since it was linear, precise, accurate, robust and selective. Moreover, this method does not require the use of organic solvents and costly equipment and materials, such as HPLC. At the same time, the turbidimetric method described in this paper takes four hours to perform, which is comparable to a physicochemical method. In addition, Student’s *t*-test showed no statistically significant difference between the proposed turbidimetric method and a previously validated HPLC method. Thus, considering the importance of microbiological methods for the analysis of antimicrobial agents, the turbidimetric method developed and validated in this work becomes a convenient alternative for the routine analysis of the quality control of this drug.
